# Study on the Structure, Magnetic Properties and Mechanism of Zn-Doped Yttrium Iron Garnet Nanomaterial Prepared by the Sol-gel Method

**DOI:** 10.3390/gels8050325

**Published:** 2022-05-23

**Authors:** Yuheng Guo, Haiyan Li, Shouqiang Li, Leilei Chen, Zhenhai Li

**Affiliations:** 1 School of Electronic Engineering, Chengdu Technological University, Chengdu 611730, China; guoyuheng111@126.com (Y.G.); gyheng1@cdtu.edu.cn (S.L.); 2 Department of Mathematics, Sichuan University, Chengdu 610065, China; 3 Urumqi Municipal Center for Disease Control and Prevention, Urumqi 830018, China; cllxiaob@163.com; 4 China’s People’s Liberation Army 32319 Troops, Urumqi 830001, China; lizhenghai_2006@126.com

**Keywords:** nanoparticles, sol-gel method, saturation magnetization, vibration modes, TG-DSC

## Abstract

To explore the effect and mechanism of bivalent ion doping on yttrium iron garnet (YIG), Zn-YIG (Zn-doped YIG) nanoparticles with a size of 60~70 nm were prepared by the sol-gel method. It was proven that Zn ion doping resulted in lattice expansion and internal stress due to crystallite size shrinkage. A Raman spectroscopic analysis proved the influence of Zn doping on the crystal structure and peak intensity by analyzing Raman vibration modes. The characteristics and chemical mechanism of mass loss and phase evolution in each temperature region were explored through TG-DSC measurements. Moreover, it was revealed that the antiferromagnetic coupling, pinning mechanisms and particle aggregation lead to coercivity, exhibiting different variation trends. A saturation magnetization (*Ms*) curve variation mechanism was further revealed, which was due to the thermal effects, super-exchange effect, and coupling effect between sub-lattices. Meanwhile, the influence of the thermal effect on *Ms* and its mechanism were explored by spin theory, and it was proven that it was mainly caused by the random arrangement of magnetic moments and thermal vibration. These results provide theoretical support for the wider application of YIG devices in microwave and high-temperature fields.

## 1. Introduction

Yttrium iron garnet (YIG) is a crystal with a special crystal structure consisting of three secondary lattices: tetrahedron, octahedron and dodecahedron, in which a Y(III) cation occupies dodecahedral (c) sites, 24 Fe(III) cations occupy tetrahedral (d) sites and 16 Fe(III) cations occupy octahedral (a) sites. The special crystal configuration gives YIG excellent ferrimagnetic properties, thermal stability and a low ferromagnetic resonance linewidth [1,2,3,4,5,6,7], so it has been widely used in the manufacture of isolators, filters, delayers and other microwave devices to create extraordinary technological and economic benefits [8,9,10,11,12,13,14].

In YIG, the magnetic moment of the nonmagnetic Y(III) ion is 0 µB, and the only one that contributes to magnetism is the Fe(III) ion (3*d*_5_) in the S state. Therefore, YIG is relatively simple from a magnetic point of view and is the basis to study other rare-earth ions doped yttrium iron garnet (RIG). In practical applications, except for the application of pure YIG with excellent performance, ion doping and substitution are often used to obtain desirable properties for some specific applications to meet various requirements [8,9,10]. The addition of Ag in YIG brought both negative permeability and permittivity. It was found that adding Zr in YIG can reduce the sintering temperature and increase its lattice constant. In addition, the incorporation of carbon in YIG resulted in a transition from insulator to conductor. 

To explore the influence of YIG doping modification, many studies have focused on ion-doped YIG and achieved good results. For instance, S. Khanra et al. explored the Mo doping effect on the magnetic properties of YIG and proved that *H*c increased with the increase in the Mo concentration. So far, there have been many studies on YIG doping modification, and remarkable results have been achieved [15,16,17,18,19]. Unfortunately, most studies focused on exploring the influence of trivalent ion doping on YIG performance, while studies on the influence of divalent ion doping on YIG are relatively few or the research is not deep enough. In particular, the deep mechanism of the influence of non-magnetic divalent zinc ion doping on the structure and properties of YIG is rarely explored. 

Based on this situation, in order to study the influence of zinc doping on the material structure, electromagnetic properties and temperature stability and to explore the physical and chemical mechanism of the influence of zinc doping on material properties, Zn-YIG nanomaterial was synthesized by the sol-gel method in this paper, and its properties and mechanism were studied. The characteristics and chemical mechanism of mass loss and phase evolution in each temperature region were explored through a TG-DSC measure. The effects of zinc doping on the crystal structure and magnetic property werestudied theoretically and experimentally. By XRD and Raman spectrum measurements, it was proventhat Zn(II) doping caused a significant influence on saturation magnetization and coercivity due to the antiferromagnetic coupling, thermal effect and coupling effect between magnetic sub-lattices (octahedral and tetrahedral). In addition, the influence of the thermal effect on *Ms* was validated by the Bragg spin theory and an *Ms*–*T* relationship measurement. In view of the relatively few published studies on the synthesis methods, properties and deep mechanism of Zinc-doped YIG, the results obtained in this paper are beneficial to the exploration and supplement for the wider use of YIG ferrite at a high temperature and high frequency.

## 2. Experiment and Characterization

Zn-YIG (Zn-doped YIG) nanoparticles were successfully prepared by the sol-gel method with different zinc contents (0, 0.01, 0.02 and 0.04 wt.%), which were denoted as Zn_0_, Zn_1_, Zn_2_ and Zn_4_, respectively. The raw materials were Y(NO_3_)_3_.6H_2_O (99.99% purity), Fe(NO_3_)_3_.9H_2_O (99% purity), Zn(NO_3_)_2_.6H_2_O (99% purity) and C_6_H_8_O_7_ (99% purity). First, Y(NO_3_)_3_.6H_2_O, Fe(NO_3_)_3_.9H_2_O and Zn(NO_3_)_2_.6H_2_O were dissolved in distilled water with a stoichiometric ratio of 3Y/5(Fe-Zn), then 0.1M citric acid (C_6_H_8_O_7_)was slowly add into the Y-Fe-Zn mixed solution. The solution was continuously stirred for 4 h. Meanwhile, by adding a small quantity of ammonium NH_4_OH, the pH value of the solution was kept at about 3. After that, the solution was heated to 80° Cuntil it transformed into gel. Thereafter, the gel was maintained for 16 h at room temperature. After this stage, the gel was dried by a heat treatment at 120 °C for 24 h and then heated up to 400 °C to burn out organic substances. Finally, the powder was sintered at 950 °C for 1.5 h in a resistance furnace and cooled down to the room temperature to obtain the Zn-YIG nanoparticles.

After synthesizing the Zn-YIG nanoparticles, phase identifications were performed using a X-raydiffraction (XRD; Tokyo, Japan). Raman spectroscopy measurements were carried out using a QE6500 spectrometer. Thermogravimetry/differential scanning calorimetry measurements (TG/DSC) were performed by a STA449F3–JUPITER/NETZSCH simultaneous thermal analyzer. The microstructures were characterized by a transmission electron microscope (TEM, JEOL JEM-2100F). The magnetic properties of all samples were characterized by a BHV-525 vibrating sample magnetometer (VSM).

## 3. Results and Discussion

### 3.1. Structural Analysis

**Figure 1:**
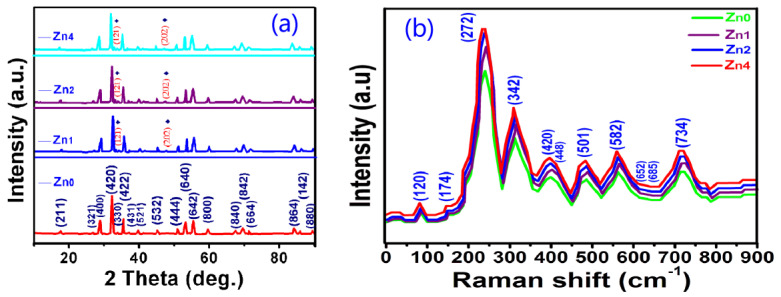
(**a**) XRD patterns and (**b**) Raman spectra of Zn-YIG nanoparticleswith different contents of zinc (0, 0.01, 0.02 and 0.04 wt.%, corresponding to Zn_0_, Zn_1_, Zn_2_ and Zn_4_).

Figure 1a depicts the XRD patterns of Zn-YIG nanoparticles prepared by the sol-gel method with different contents of zinc (0, 0.01, 0.02 and 0.04 wt.%), corresponding to Zn_0_, Zn_1_, Zn_2_ and Zn_4_). It can be seen that no impurity phase was observed in the samples, except for small amount of orthoferrite phase (CoD 96-210-1387), marked by ♦.

To supply the XRD results and confirm the occupied sites of Zn ion, Raman spectroscopy measurements were carried out. Figure 1b shows the Raman spectra of the Zn-YIG samples measured in the normal condition. In a typical garnet crystal structure, according to group theory, it is predicted that the first-order Raman spectrum contains 14T_2g_ + 3A_1g_ + 8E_g_ modes, while a 17T_1u_mode should appear in the infrared spectrum. However, only 11 of the above-mentioned 25 theoretically predicted Raman modes were detected in the Zn-YIG samples. This may be due to two factors: one is that the intensity of some modes are too weak to be observed, and the other is that there is an accidental degeneracy factor of several vibrational modes at room pressure. The degeneracy of these modes is derived from two aspects: (1) the small wave vectors with every mode splitting into a singly optical degenerate longitudinal vibration and (2) the double optical degenerate transverse vibration under the influence of long-range electrostatic forces. From Figure 1b, it can be seen that the curves of all samples are roughly the same, except for the relative peak intensities. The Raman peaks corresponding to the 120, 174 and 272 sites were derived from the cation translationalmotions. For pure garnet at the 272 site, the T_2g_ mode stems from the characteristic translational vibration mode of a cation in the dodecahedral and tetrahedral sites, and the sharp feature of the T_2g_ mode is derived from the Franck Condon principle. For Zn-YIG samples, vibration mode frequency changes little, but peak intensity changes slightly, which may be due to the influence of larger zinc ion doping. In addition, by analyzing the Raman spectrum, we can also obtain these results: (1) the peaks at the 685 and 420 sites come from the combination of modes T_2g_ and E_g_, (2) the peaks at the 448 and 341 sites belong to the combination of modes A_1g_ + T_2g_ and modes A_1g_ + E_g_, (3) the peaks at the 120, 174 and 582 sites result from mode T_2g_, and (4) the peak at the 652 site stems from phonon mode E_g_.

**Figure 2:**
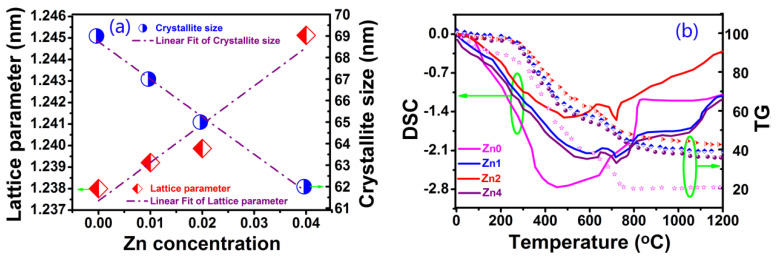
(**a**) The variation of lattice constant and crystallite size and (**b**) TG—DSC curves of dried gel.

Figure 2a shows the variations in the crystallite sizes and lattice constant of YIG nanoparticles. It can be observed from Figure 2a that lattice constant shows a linear increase from 1.23800 to 1.24512 nm with the increase in Zn content from *x* = 0 to 0.04. The reason may be that the doping of a larger zinc ion causes crystal expansion. With the increase in Zn content, more and more Zn ion doping induced internal stress and strain, which hindered crystallite growth. As a result, the crystallite sizes of samples showed a tendency to decrease from 69 to 61nm when the Zn content increases from *x* = 0 to 0.04, which is in agreement with the size calculated by the Scherrer equation (D = Kλ/βcosθ, where D is the average grain size, K is the grain shape factor, λ is the X-ray wavelength, β is the intrinsic increased width, and θ is the Bragg angle).

**Figure 3:**
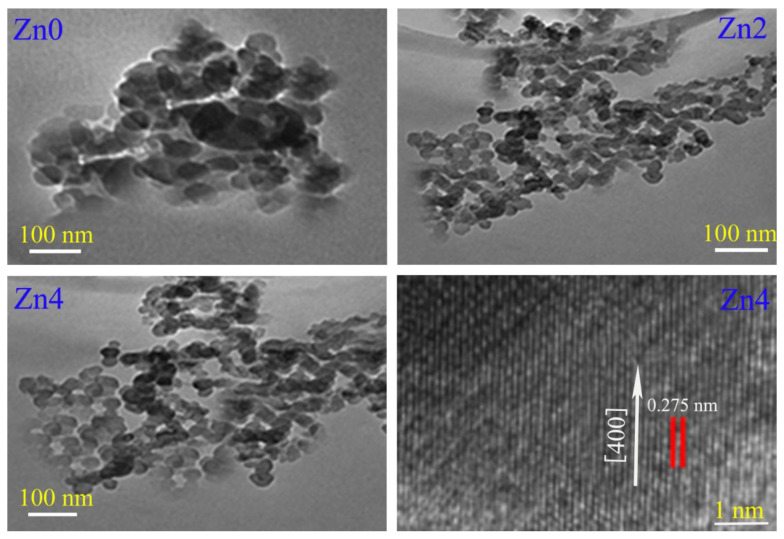
TEM and HRTEM images of Zn_0_, Zn_2_ and Zn_4_ samples.

Figure 3 shows TEM and HRTEM images of Zn_0_, Zn_2_ and Zn_4_ samples. It is easy to discern from Figure 3 that all particles were roughly spherical with a particle size distribution in the range of 60~70 nm, which is in good agreement with the values calculated by the Scherer equation. Compared with sample Zn_0_, samples Zn_2–4_ had smaller particle sizes and more serious agglomeration. This is because with the increase in zinc content more and more zinc ion doping caused internal stress and strain, impeding the growth of crystals and reducing the grain size of samples from 69 nm to 61 nm, which is consistent with the previous analysis. The interplanar distance calculated from HRTEM was 0.275 nm, which is consistent with the interplanar spacing of the (400) crystal plane. Although the spherical particles in Figure 3 were treated by an ultrasonic, the agglomeration phenomenon was still serious, which may have been caused by alloying and cold welding between particles during the milling process, especially high-speed ball milling to particles. These agglomeration phenomena may have been derived from four aspects: (1) due to the large surface area and high surface energy of nanoparticles, particles were in an unstable energy state, which eventually lead to particle agglomeration to reach a relatively stable state; (2) when particles were refined to a nanometer scale, a large number of positive and negative charges accumulated on the surface of particles, and nanoparticle shape was generally irregular, which made articles extremely unstable, leading to particle agglomeration due to the accumulation of surface charges; (3) because the distance between nanoparticles was very short, the VanDer Walls force between particles was much larger than their own gravity, so particles were easy to attract to each other and cause agglomeration; and (4) the interaction of a large number of hydrogen bonds and chemical bonds on the surface of nanoparticles also easily caused adsorption phenomenon between particles, which eventually lead to particle agglomeration.

The thermogravimetry (TG) and differential scanning calorimetry (DSC) measures were performed for all samples, and Figure 2b shows the phase evolution and thermal analysis curves (DSC-TG) of dried gel. The TG curves may be divided into four regions: (a) In the 0~250 °C region, the dried gelslostabout 5% of their weight in the gel dehydration process; (b) In the 250~700 °C region, the average mass loss reached 40%, stemming from organic material burning from reactions and citrate molecule rupture; (c) In the 700~800 °C region, the mean mass loss was about 14% due to the formation of orthoferrite YIG crystals and the subsequently major cubic YIG; (d) In the 800~1200 °C region, samples showed different mass loss behaviors. For the Zn_0_ sample, mass loss reached 20% at 800 °C without any mass loss from 800 to 1200 °C, but samples Zn_1_, Zn_2_ and Zn_4_ showed a tendency to slow the decline in mass loss from 800 to 1200 °C. The reason is that the incorporation of Zn^2+^ in the YIG structure leads to a new phase formation. The crystal chemical environment of YIG is changed to adapt to new chemical bonds and lattice deformation due to zinc ion doping, so it results in different mass losses for different Zn-doped samples. To verify the formation of a new phase from 800 to 1300 °C, we performed DSC measurements for all samples, and the results show apparent new phase formation above 1100 °C, especially for the samples with higher Zn contents. Further research can prove that this is due to the formation of a ZnFe_2_O_4_ phase.

Figure 3 shows TEM and HRTEM images of Zn_0_, Zn_2_ and Zn_4_ samples. It is easy to discern from Figure 3 that all particles were roughly spherical with a particle size distribution in the range of 60~70 nm, which is in good agreement with the values calculated by the Scherer equation. Compared with sample Zn_0_, samples Zn_2–4_ had smaller particle sizes and more serious agglomeration. This is because with the increase in zinc content more and more zinc ion doping caused internal stress and strain, impeding the growth of crystals and reducing the grain size of samples from 69 nm to 61nm, which is consistent with the previous analysis. The interplanar distance calculated from HRTEM was 0.275 nm, which is consistent with the interplanar spacing of the (400) crystal plane. Although the spherical particles in Figure 3 were treated by an ultrasonic, the agglomeration phenomenon was still serious, which may have been caused by alloying and cold welding between particles during the milling process, especially high-speed ball milling to particles. These agglomeration phenomena may have been derived from four aspects: (1) due to the large surface area and high surface energy of nanoparticles, particles were in an unstable energy state, which eventually lead to particle agglomeration to reach a relatively stable state; (2) when particles were refined to a nanometer scale, a large number of positive and negative charges accumulated on the surface of particles, and nanoparticle shape was generally irregular, which made articles extremely unstable, leading to particle agglomeration due to the accumulation of surface charges; (3) because the distance between nanoparticles was very short, the VanDer Walls force between particles was much larger than their own gravity, so particles were easy to attract to each other and cause agglomeration; and (4) the interaction of a large number of hydrogen bonds and chemical bonds on the surface of nanoparticles also easily caused adsorption phenomenon between particles, which eventually lead to particle agglomeration.

### 3.2. Magnetic Properties

**Figure 4:**
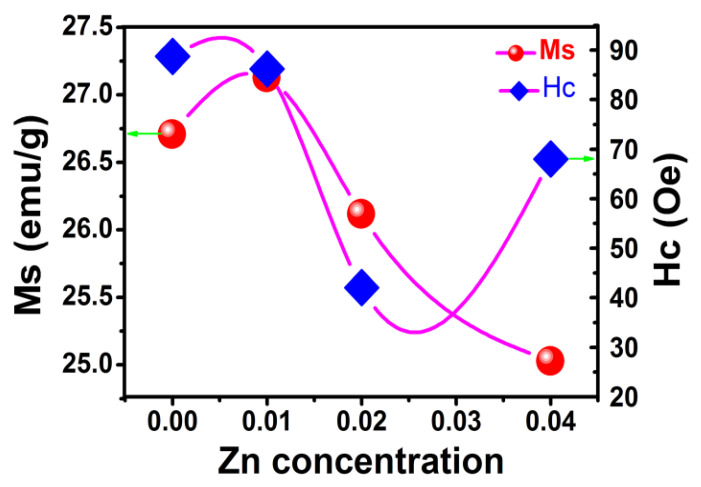
The variation in saturation magnetization and coercivity.

Figure 4 shows the variation of saturation magnetization (*Ms*) and coercivity (*H*c) with zinc content in Zn-YIG. Except for Zn_2_, coercivity presents a general declining trend for samples Zn_0_, Zn_1_ and Zn_4_. This is because zinc ion doping reduces antiferromagnetic coupling of iron ions in an octahedral lattice, forming a new pinning mechanism and changing the local exchange coupling constant, which opens a new way for the magnetization reversal of doped samples. Consequently, coercivity must be modified. In addition to the pinning mechanism of the magnetic moment, coercivity is related to particle size, stomata, shape and formation. The coercivity of sample Zn_2_ was obviously lower than that of other samples. It may have been because the Zn_2_ sample presented more agglomeration than samples Zn_0_, Zn_1_ and Zn_4_, which changed the particle distribution, formation and morphology, and ultimately lead to a significant decrease in coercivity.

**Figure 5:**
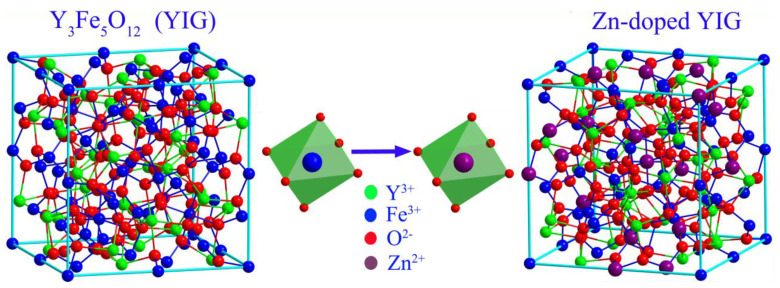
Crystal structure evolution model.

As can be seen in Figure 4, due to the influence of Zn(II) doping and thermal effect, the saturation magnetization curve experienced a trend from small to large and then slowly decreased, which can be explained in combination with a crystal structure evolution model shown in Figure 5. For YIG material, magnetic ordering originates from the super-exchange interaction between tetrahedral sites (occupied by a nonmagnetic Y ion) and octahedral sites (occupied by an Fe ion). Due to the doping of the larger zinc ion, antiferromagnetic coupling was reduced, resulting in an increase in net magnetic moment and magnetization. Therefore, saturation magnetization (*Ms*) of sample Zn_1_ was higher than for sample Zn_0_. However, with a further increase in Zn content, saturation magnetization showed a tendency to slowly decline due to the weakening of magnetic coupling between two sub-lattices. For YIG material, the total magnetic moment relies on the coupling effect between two magnetic sub-lattices (octahedral and tetrahedral). Due to the dual influences of the thermal effect and increased zinc content, magnetic coupling between sub-lattices is greatly diminished, which must lead to a decrease in net magnetic moments and total magnetic moments. Hence, saturation magnetization showed a decreasing trend with the increase in Zn content. To further explore the influence of the thermal effect on *Ms*, the temperature dependence of the magnetic saturation measurement was evaluated.

**Figure 6:**
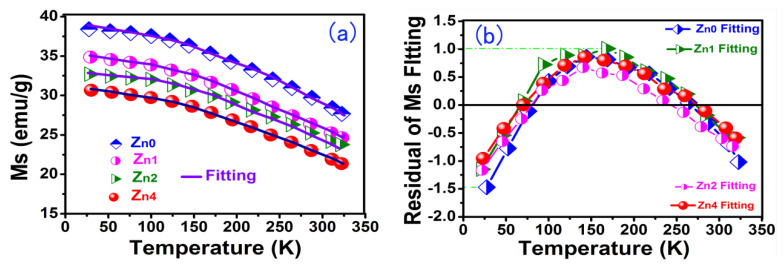
(**a**) The temperature dependence of magnetic saturation for Zn—YIG samples and (**b**) regular residual of fitting temperature dependence of magnetic saturation.

Figure 6a shows the temperature dependence of magnetic saturation for the Zn-YIG samples. With the decrease in temperature, the saturation magnetization curves showed an increasing tendency. When temperature was relatively high, magnetic moments were randomly arranged, resulting in a decrease in the total magnetic moment, which inevitably lead to a decrease in saturation magnetization. As temperaturedecreased, the randomization degree of the magnetic moment in relation to the applied magnetic field was reduced due to the weakened thermal vibration, which gave rise to a decrease in the total magnetic moment, so *Ms* was enhanced as the temperature dropped. The relationship between temperature and *Ms* can be described by the Bloch law (1):

(1) $$M_{S}{}_{\left(T\right)}=M_{s}{}_{\left(0\right)}-\frac{bM_{s(0)}T^{n}}{T_{C}{}^{n}}$$

where *b, n* and *T_C_* are the Bloch’s constant, Bloch exponent and Curie temperature, respectively. *M*_*S*(0)_ is the magnetic saturation of 0 K, and *M_S(T)_* is dominated by the spin wave fluctuation.

We fit Equation (1) using the test data of Figure 6a, and the fitting parameters are listed in Table 1. In Table 1 it may be seen that Adj.R-Square fluctuates between 0.973 and 0.991, which is approximately equal to 1. The regular residual value fluctuates between +1.012 and −1.487, as shown in Figure 6b. Therefore, the experimental result is highly consistent with the Bloch law theoretical analysis, which fully demonstrates the influence of the thermal effect on *Ms*. Furthermore, as expected, the saturation magnetization (*Ms*) in Table 1 shows a downward trend with increasing Zn content, which is due to the decrease inthe net magnetic moment caused by the non-magnetic Zn(II) ion.

## 4. Conclusions

Zn-YIG nanoparticles with different zinc contents were successfully synthesized by the sol-gel method. XRD, Raman spectroscopy, TG/DSC, SEM and VSM measurements were performed to characterize and explore the influence of zinc doping on the structure and properties and its deep mechanism.

The main findings in this work include:

(1) XRD verified the inverse relationship between the lattice constant and crystallite, which is attributed to lattice expansion due to the doping of the larger zinc ion and the crystal size shrinkage caused by internal stress and strain.

(2) Raman spectroscopy measurement revealed the structural relationship of Zn-YIG crystals. By analyzing the vibration modes and peak positions, it was found that the cation characteristic translational vibration mode, T_2g_ mode sharp feature, and larger size Zn(II) ion doping perturbed peak intensities, resulting in only lattice parameter shift but no change in the crystal structure.

(3) TG measurement curves were divided into four regions, and the mass loss characteristics and chemical mechanism of each region were explored. Meanwhile, DSC was performed and confirmed that the formation of a new phase (ZnFe_2_O_4_) was the basic reason for the different mass loss behaviors at 800~1200 °C.

(4) It was found that antiferromagnetic coupling, the magnetic moment pinning mechanism and particle aggregation caused by Zn ion doping made coercivity increase first and then decrease with the increase in Zn content.

(5) The mechanism of saturation magnetization changing with zinc content was explored, which can be attributed to the super-exchange interaction between tetrahedral sites, the coupling effect between two magnetic sub-lattices and the thermal effect.

(6) The influence of the thermal effect on *Ms* was confirmed by measuring the temperature dependence of magnetic saturation and fitting the Bloch law using the pin wave theory. It was proven that the thermal effect was mainly caused by thermal vibration and the randomization of the magnetic moment arrangement.

The obtained results are of great significance to ferrite application in high-temperature and high-frequency environments.

## Figures and Tables

**Table 1:** Fitting results of saturation magnetization using the Bloch law.

Samples	Zn_0_	Zn_1_	Zn_2_	Zn_4_
Adj.R-Square	0.991	0.973	0.986	0.988
Regular Residual value	[−1.487, 0.978]	[−1.246, 1.012]	[−1.245, 0.728]	[−1.002, 0.976]
*Ms*	39.231	36.214	34.213	33.356
*H*c	44.1	27.2	24	37
*n*	1.9376	1.8762	1.8632	1.9543
b.(*T*c)^-n^	4.9123	7.9186	8.4212	4.7839

Adj. R-Square: a modified version of R-squared that has been adjusted for the number of adjustable parameters in the model.
